# Physiology of the Right Ventricle Across the Lifespan

**DOI:** 10.3389/fphys.2021.642284

**Published:** 2021-03-02

**Authors:** Kathleen C. Woulfe, Lori A. Walker

**Affiliations:** Division of Cardiology, Department of Medicine, University of Colorado Anschutz Medical Campus, Aurora, CO, United States

**Keywords:** right ventricle, pediatric, aging, physiology, hemodynamics

## Abstract

The most common cause of heart failure in the United States is ischemic left heart disease; accordingly, a vast amount of work has been done to elucidate the molecular mechanisms underlying pathologies of the left ventricle (LV) as a general model of heart failure. Until recently, little attention has been paid to the right ventricle (RV) and it has commonly been thought that the mechanical and biochemical properties of the RV are similar to those of the LV. However, therapies used to treat LV failure often fail to improve ventricular function in RV failure underscoring, the need to better understand the unique physiologic and pathophysiologic properties of the RV. Importantly, hemodynamic stresses (such as pressure overload) often underlie right heart failure further differentiating RV failure as unique from LV failure. There are significant structural, mechanical, and biochemical properties distinctive to the RV that influences its function and it is likely that adaptations of the RV occur uniquely across the lifespan. We have previously reviewed the adult RV compared to the LV but there is little known about differences in the pediatric or aged RV. Accordingly, in this mini-review, we will examine the subtle distinctions between the RV and LV that are maintained physiologically across the lifespan and will highlight significant knowledge gaps in our understanding of pediatric and aging RV. Consideration of how RV function is altered in different disease states in an age-specific manner may enable us to define RV function in health and importantly, in response to pathology.

## Introduction

While the majority of studies in cardiac dysfunction focus on the left ventricle (LV), it is clear that function and regulation of the right ventricle (RV) are distinct from the LV. Understanding normal physiological differences between the LV and RV is essential to elucidating unique aspects of pathophysiology in each ventricle. Even more importantly, age-specific regulation of cardiac structure and function is emerging as key distinctions in cardiac physiology. This review considers the physiology of the RV across the lifespan with the goal of emphasizing the need to improve our understanding of mechanisms unique to each ventricle at each life stage in order to tailor therapies and improve patient outcomes.

It is important to remember that the RV derives from unique progenitor cells during development; the LV cardiomyocytes arise from the heart tube, whereas RV cardiomyocytes derive from precursor cells in the anterior heart field (Zaffran et al., 2004). This developmental difference in cardiomyocyte origin suggests that chamber-specific cardiomyocytes may have unique properties based on functional postnatal needs. However, it is not clear if the unique embryologic origin or simply a different pressure and volume environment postnatally are responsible for the differences in RV cardiomyocyte function.

## Right Ventricular Functional Analysis

Clinically, echocardiography is the most commonly used modality for quantifying *in-vivo* cardiac function and echocardiographic analysis of LV function is robust and reliable. Analysis of RV function, though, is less reliable, due to its sub-sternal position and its complex geometry. However, tricuspid annular plane systolic excursion (TAPSE), a measure of RV longitudinal shortening, has been shown to be an accurate measure of RV contraction and is the most commonly used parameter for quantifying RV function (Schneider and Binder, 2018). TAPSE has been found to correlate well with RV ejection fraction and systolic function in adults (Ueti et al., 2002). However, Koestenberger et al. (2009) have shown that in children, there is an age-dependent increase in TAPSE, suggesting that RV function in children continues to mature throughout childhood (Koestenberger et al., 2011; Koestenberger and Ravekes, 2012). Interestingly, TAPSE seems to peak by the second or third decade of life and slowly declines through adulthood into aging (Innelli et al., 2009). Furthermore, the usefulness of TAPSE in describing RV function is well-described in both RV failure (Ghio et al., 2000) and LV failure (Kjaergaard et al., 2009).

While traditional doppler echocardiography remains the standard for cardiac functional analysis, other non-invasive measures of RV function including magnetic resonance imaging (MRI) and two-dimensional speckle tracking echocardiography are emerging as important tools in the early identification of RV dysfunction in patients of all ages (Ozturk et al., 2017; Khairat et al., 2019) and cardiac MRI is able to distinguish tissue characteristics, allowing for quantification of myocardial fibrosis which has been linked to age-related cardiac dysfunction (Liu et al., 2013). Invasive hemodynamic measurements using conductance catheters inserted into the RV provides the most accurate analysis of RV function but are less commonly used due to the invasive nature, high cost and difficulty in obtaining highly reproducible data which is largely attributable to difficulties with accurate catheter placement (Ullah et al., 2020).

Several methods exist for quantifying *ex-vivo* cardiac muscle function and we have recently reviewed these methods in detail (Knight et al., 2021). *Ex-vivo* cardiac muscle function can be assessed from the whole heart level using Langendorff preparations to the level of sarcomere or even of isolated protein-protein interactions. One of the most commonly used preparations for assessing cardiac muscle function is the trabeculae (or papillary muscle) preparation. This multicellular preparation allows for analysis of several mechanical properties (force, calcium sensitivity, and cooperativity) and provides information about the impact of the extracellular matrix on these mechanical measurements. Isolated cardiomyocyte preparations allow analysis of similar parameters in the absence of the influence of the extracellular matrix. Experiments using the cardiac muscle preparation or the isolated myocyte preparation can be conducted on intact or skinned preparations, the latter proving direct analysis of calcium sensitivity of the preparation. Each analysis technique has both strengths and weaknesses and often need to be used in conjunction with one another to create a comprehensive view of overall cardiac function.

## Right Ventricular Physiology in Adults

**Figure 1:**
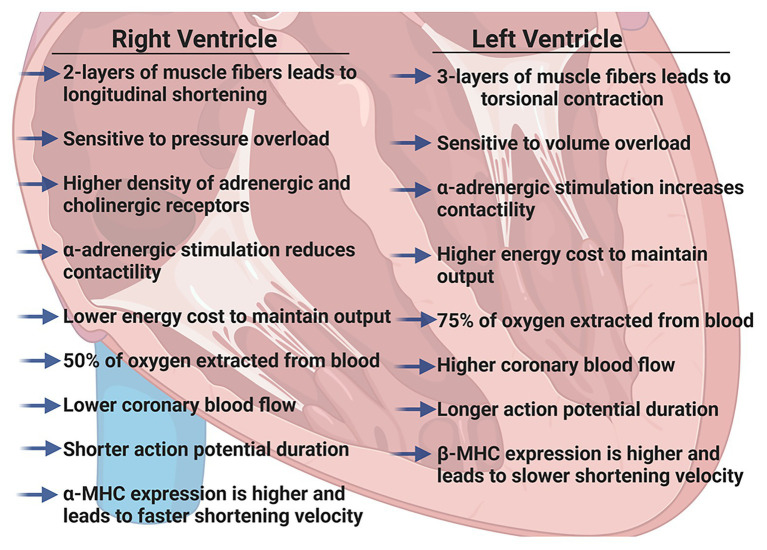
Summary of physiologic differences between the right ventricle (RV) and the left ventricle (LV) in adult hearts. Figure created using BioRender.

**Figure 2:**
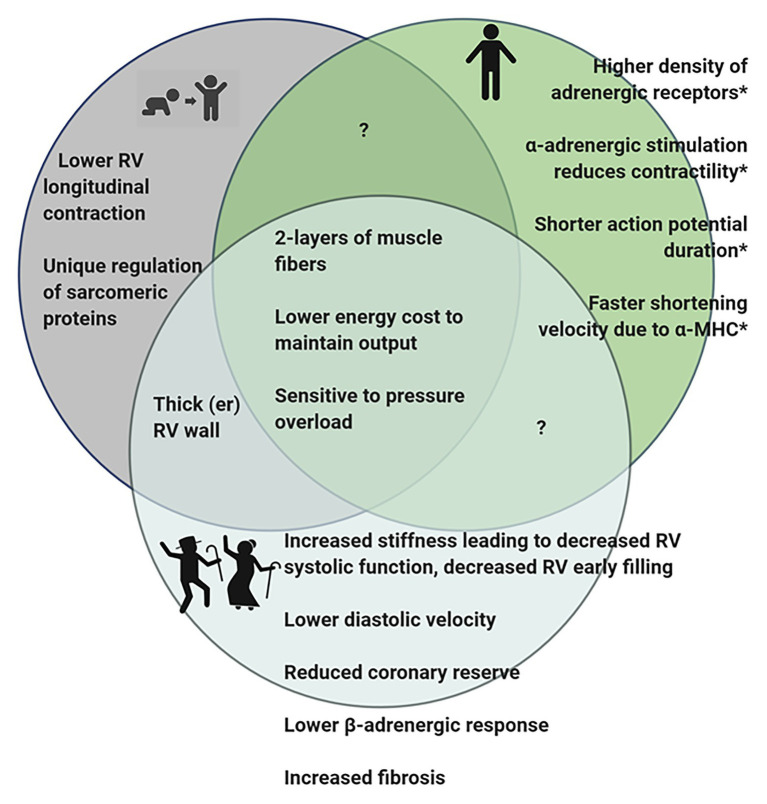
Diagram summarizing observed differences in the RV of pediatric, adult, and geriatric hearts. ^*^Some of the differences observed in adult RV have not been studied in juvenile or aging hearts and it is therefore, not known if these features are present in the hearts at different stages of life. Figure created using BioRender.

We have previously described the unique structural and functional differences of the RV in the adult (Walker and Buttrick, 2009, 2013). Here, we will briefly summarize the important RV differences in *in vivo* function (Figure 1), molecular and biochemical signaling and adaptations to pathologic stresses in order to effectively highlight differences between pediatric, adult and aging RV (Figure 2).

### *In vivo* Function of the Healthy Adult RV

The RV is a thin-walled, crescentic-shaped chamber that is coupled to the low pressure pulmonary circulation (Ho and Nihoyannopoulos, 2006). The muscle fibers in the RV are generally aligned in two layers; a superficial layer arranged circumferentially and a deeper longitudinal layer. Due to this arrangement, the RV has a more limited contractile motion, usually seen as longitudinal shortening rather than the wringing, torsional contraction of the LV (Dell’Italia, 1991; Ho and Nihoyannopoulos, 2006). Because the adult RV wall thickness is considerably less than LV wall thickness, the RV is more load-dependent and acutely, even modest increases in pulmonary vascular resistance (PVR) can result in significant declines in RV cardiac output. However, with chronic pressure overload (i.e., pulmonary hypertension) RV remodeling and hypertrophy can occur as an initial adaptive response that precedes RV thinning and failure (van der Bruggen et al., 2017).

Regulation of RV contraction differs from that of the LV in several ways. Both adrenergic and cholinergic receptor densities are slightly higher in the RV than LV (Bristow et al., 1992) and Wang et al. (2006) have suggested antithetical effects of adrenergic stimulation; α-adrenergic stimulation of the RV reduces contractility but increases contractility of the LV. Additionally, lower afterload pressures in the pulmonary circulation lead to a lower work of contraction in the RV and the energy cost of maintaining the same cardiac output as the LV is only ~20% that of the LV (Reddy and Bernstein, 2015).

Oxygen supply to each ventricle occurs through left and right coronary arteries. High systolic contractile forces in the LV create compressive forces on the left coronary arteries such that the majority of coronary blood flow occurs during diastole, whereas the lower contractile pressures in the RV allow for more continuous coronary artery blood flow. However, coronary blood flow is directly related to perfusion pressure and inversely related to PVR and in the adult heart, it has been well-established that RV coronary blood flow is lower than LV coronary blood flow (Crystal et al., 1991; Crystal and Pagel, 2018). Furthermore, the RV extracts a lower proportion of O_2_ supplied (~50% compared to ~75% in the LV), providing a more substantial O_2_ reserve. The RV can quickly adapt to increases in O_2_ demand by increasing coronary blood flow and increasing O_2_ extraction. Clinically, this has recently been elegantly demonstrated in healthy adults using RV conductance catheters to collect real-time pressure-volumes loops (Cornwell et al., 2020). The authors found that resting RV energy expenditure was markedly lower than that of the resting LV and that RV energy expenditure increased dramatically with exercise to levels comparable with the resting LV.

### Molecular and Biochemical Properties of the Adult RV

Functionally, there are several reports highlighting differences between isolated muscles (or cardiomyocytes) from the RV and LV. It has been shown that in tissues isolated from the RV, force production (Brooks et al., 1987) and sarcomere shortening (Kondo et al., 2006) are reduced, but that shortening velocity is greater (Harding et al., 1990). In accordance with these findings, the action potential duration has been shown to be shorter in RV cardiomyocytes and calcium transients are reduced when compared to LV cardiomyocytes (Kondo et al., 2006). Furthermore, the RV expresses significantly more α-myosin heavy chain than the LV in both rats (Brooks et al., 1987) and rabbits (Litten et al., 1985). However, while these molecular differences may correlate with unique RV function and begin to describe the differences between the RV and LV, the molecular and cellular underpinnings of these fundamental differences are still not completely understood.

As described above, chamber-specific energy utilization is different with the RV demonstrating lower energy expenditure. Interestingly, transcriptome analysis of metabolic pathways demonstrated significant differences in gene expression between the RV and LV (Drake et al., 2011). While differentially expressed genes spanned a wide variety of cellular pathways, the authors describe 77 differentially expressed genes associated with carbohydrate, lipid and protein metabolism. These differences in metabolic gene expression have been found both in the healthy heart and in disease, suggesting chamber-specific molecular regulation of energy metabolism.

Studies aimed at elucidating differences in ventricular-specific responses to volume and pressure overload have further highlighted cellular differences between the ventricles. For example, the RV upregulates significantly more growth factors and has a more robust fibrotic response than the LV in response to volume overload (Modesti et al., 2004), several microRNAs (miRNA 28, 148a, and 93) shown to be decreased in LV failure are increased in an animal model of RV failure (Reddy et al., 2012), and atrial natriuretic peptide, a molecular marker of cardiac dysfunction, is only minimally expressed in adult RV (Drexler et al., 1989; Raizada et al., 2001; Broderick et al., 2010) compared to LV.

Additionally, several studies have demonstrated both ventricular and age-related differences in adrenergic receptor expression. In one rat model of catecholamine excess following burn injury (Guillory et al., 2017), investigators found that both β_1_-and β_2_-adrenergic receptors were significantly less abundant in the RV of control animals and that there was a significant upregulation of the β_2_-adrenergic receptor in the RV of injured animals in contrast to unchanged receptor expression in the LV. Similarly, in the isolated perfused heart preparation, chronic infusion of norepinephrine increased RV systolic pressure by more than 100%, whereas it increased LV systolic pressure by less than 20% (Irlbeck et al., 1996). This differential responsiveness to catecholamines may be due in part to the biventricular differential expression of adrenergic receptors. Interestingly, differences in adrenergic receptor density have been demonstrated in pediatric heart compared to adult hearts (Miyamoto et al., 2014), and in one randomized control trial of beta-blockade using carvedilol in children, there was no benefit compared to control (Singh and Preuss, 2020), in contrast with adult response to carvedilol (Singh and Preuss, 2020).

Lastly, another important consideration underscoring that the RV is molecularly unique from the LV is the response, or lack of response of the RV to therapies used to treat LV dysfunction. Several studies have shown that RV from failing patients respond to therapies differently than LV from failing patients (Zimmer, 1994; Irlbeck et al., 1996; Padrini et al., 1996, 1999). However, *in vivo*, it is extremely difficult to distinguish the direct pharmacologic effects of therapies on the RV as many of these therapeutic agents alter either LV function or the pulmonary vasculature and changes in either of these can lead to adaptive changes in the RV. Therefore, it is critical to study the molecular response of the RV to various therapies. As discussed earlier, there is a paucity of data directly assessing RV molecular differences either physiologically or in disease; similarly, there are very few studies that examine the individual response of the RV to therapies. However, in one study, Padrini et al. (1999) demonstrated that the RV explanted from patients with idiopathic cardiomyopathy and coronary artery disease has a decreased response to ouabain compared to the matched LV.

Additionally, pharmaceuticals targeted to the renin-angiotensin-aldosterone system (RAAS) are often used to improve LV function in heart failure but have failed to demonstrate improvement in RV function (Lester et al., 2001; Dore et al., 2005; Therrien et al., 2008; Ameri et al., 2016; Amsallem et al., 2018). Conversely, several studies in animal models of pulmonary arterial hypertension suggest that RAAS blockade may improve RV function (Rouleau et al., 2001; Okada et al., 2008, 2009), but as highlighted above, it is difficult to distinguish the direct effects of these therapies on RV function from secondary effects related to the impact the therapies had on the pulmonary vasculature. Importantly, there seem to be age-specific differences in the effectiveness of RAAS-blockade in the LV suggesting once again that it is critical to understand the impact of these therapies on the RV across the lifespan.

There is an interesting contrast in therapies such as phosphodiesterase inhibitors (PDEi) that have been found to be effective and safe in pediatric patients with heart failure but not as safe in adults (reviewed; Miyamoto et al., 2018). Moreover, PDE5i may be beneficial in improving RV function but it difficult to determine if this improvement is due to direct cardiomyocyte effects or simply improvement in pulmonary vascular function (Nagendran et al., 2007; Garcia et al., 2018). It is not clear if PDEi are beneficial in RV function in adults. In fact, in an animal model of pulmonary arterial banding, PDE5 inhibition increased mortality and did not improve right ventricular function (Andersen et al., 2013).

Overall, more studies are needed to determine how therapies impact the function of RV across the lifespan. Importantly, these differences indicate that physiologic mechanisms distinct to the RV play an important role in response to therapies.

## Right Ventricular Physiology in Children

Even less is known about differences in RV molecular and biochemical properties in pediatric RV. In fact, it has been assumed that pediatric hearts are simply smaller versions of adult hearts. However, it is becoming increasingly clear that there are key differences in regulation and molecular function of the RV in pediatric hearts.

### *In vivo* Function of the Healthy Pediatric RV

Since pediatric development encompasses the time from birth to 18 years of age, this represents a continuum of physical changes; therefore, it is important to consider the various functions that the RV is called upon to complete at different life stages. It is likely that the changing demands occurring over the lifespan underlie the unique function of the RV. In the developing fetus, the RV works together with the LV to provide systemic circulation and ejects blood at relatively high pressures, largely bypassing the nonventilated lung through the patent foramen ovale and ductus arteriosus. While the systemic pressures during this time are low, the RV wall is thick and the force the RV produces is equivalent to LV force (Haddad et al., 2008; Sanz et al., 2019). It is clear that during fetal development that the RV and LV are capable of developing the same force and are physiologically similar; moreover, in congenital conditions where patients have a single right ventricle, the RV is able to temporarily meet the systemic pressure challenges until surgical interventions are in place (Sable et al., 2011; Garcia et al., 2020). However, over time the single RV is prone to failure suggesting that the RV cannot fully adapt to adequately serve the high pressure systemic circulation. This may be due to intrinsic differences in the RV structure and cardiomyocyte function.

At birth, the RV and LV wall thickness is approximately the same (Firpo et al., 2001). However, birth results in a major change in the pressures to which the RV is exposed as PVR and pulmonary artery pressures rapidly lower at birth. Once this occurs, the RV is essentially unloaded, leading to increased compliance and thinning of the RV wall. By early childhood, the structure of the RV is similar to that of an adult (Haddad et al., 2008).

### Molecular and Biochemical Properties of Pediatric RV

Little is known about whether RV molecular function differs between pediatric and adult hearts. Once again, there is some assumption of underlying age differences as the RV undergoes radical changes at birth. In this sense, it is reasonable to presume that different molecular function parallels the structural differences between pediatric and adult cardiomyocytes and that RV cardiomyocytes may retain unique features through childhood. One important finding suggests that the RV from juvenile Yorkshire piglets exposed to acute or chronic pulmonary banding inadequately adjusts substrate oxidation resulting in an energy imbalance (Kajimoto et al., 2018, 2019). Further studies have also shown that the metabolic response of the RV is decreased compared to the LV response (Kajimoto et al., 2017). This is in line with other studies in the LV that also demonstrate a difference in metabolism in young hearts compared to adult (Portman et al., 1989). These findings demonstrate a critical need to understand molecular mechanisms in pediatric RV.

In addition to indications that RV metabolism is unique in an age dependent manner, a study defining RV failure in a neonatal calf model of pulmonary hypertension suggests that although sarcomeric proteins undergo post-translational modifications consistently reported in the failing LV, the mechanical response is distinctly different in the RV (Walker et al., 2011). This mechanical difference may be reflective of the fact the RV is optimized to handle volume overload conditions but not as readily able to respond to pressure overload challenges. However, it is not known if the mechanical differences persist in adult RV or if this is another unique age-dependent difference in RV function.

Similar to what has been reported in adult LV failure, RV dysfunction has been reported in severe cases of LV failure in children (Agha et al., 2017). Importantly, guideline approved therapies that are efficacious in adults with dilated cardiomyopathy are not as effective in children with dilated cardiomyopathy (Shaddy et al., 2007; Hsu et al., 2010; Kantor et al., 2010) suggesting that there are age-specific molecular mechanisms at play (Agha et al., 2017). Based on the fact that there are age-specific molecular differences that impact the failing LV, it is highly likely that there are also unique molecular features of the RV that differ across the lifespan and make it imperative to study RV (patho) physiology in all ages.

## Right Ventricular Physiology in Aging Adults

As with pediatric hearts, the function of the aging RV has often been assumed to be the same as adult function. However, there are changes associated with physiologic aging that impact RV function and warrant consideration.

### *In vivo* Function of the Healthy Geriatric RV

Similar to observations in the LV, studies have demonstrated decreased RV systolic function, decreased early RV filling during diastole, and lower diastolic velocities in the RV associated with aging (Klein et al., 1999; Henein et al., 2014; Kachenoura et al., 2020). Additionally, the RV wall thickness increases with age. These changes suggest that ventricular stiffening increases with age in the RV as it does in the LV. In agreement with these studies, Addetia et al. (2018) demonstrated differences in the echocardiogram parameters that suggest that as humans age the RV is stiffer and produces a less “bellows-like” movement. Animal models validate that aging infers significant changes in RV systolic and diastolic function (Kajstura et al., 1996; Kuo et al., 2018). Furthermore, the PVR increases with age, increasing the afterload on the RV, which may account for RV wall thickening with age (Henein et al., 2014). Lastly, as mentioned above, the RV has a higher coronary reserve. However, in the RV of aging rats, RV coronary flow is reduced and the resistance is increased, suggesting that the coronary reserve may be reduced in the aging RV (Hachamovitch et al., 1989).

### Molecular and Biochemical Properties of Geriatric RV

While a number of studies have observed functional changes in the aging RV, fewer studies have described molecular and biochemical changes the aging RV. One study in rats suggests that resting tension is higher and duration of contraction is shorter in right papillary muscles compared to papillary muscles isolated from the LV. However, in aged rats, the dynamics of papillary muscles from the RV were resistant to changes that occurred in the LV papillary muscles (Anversa et al., 1989) and RV papillary muscles from aging rats were more protected from myocardial injury.

Interestingly, several studies have investigated the impact of aging on β-adrenergic receptor density and activity between the RV and the LV. Studies in aging rats demonstrated no differences in RV β-adrenergic receptor density (Kusumoto et al., 1994); while a study in turkeys concluded that overall β-adrenergic receptor density decreased with age in both LV and RV (Hoffmann et al., 2016). This is in line with a study by White et al. (1994) that reported a decreased density of β -adrenergic receptors in the RV of aging donor hearts. In addition, systolic response of RV trabeculae from older patients was decreased in response to isoproterenol (White et al., 1994).

Extracellular collagen is increased in the RV of aging rats (Eghbali et al., 1989; Thomas et al., 2001). Interestingly, Eghbali et al. (1989) reported that RV accumulation of collagen in 22-month-old rat hearts is greater than collagen accumulation in the LV, and Thomas et al. (2001) demonstrated that exercise training in aging rats can decrease collagen deposition in the LV but not the RV, suggesting that there are alternative mechanisms for regulation of extracellular matrix between the two ventricles.

In aging LV, studies have demonstrated that myocytes undergo cell death. However, it is unclear if similar myocyte loss occurs in the RV. Phaneuf and Leeuwenburgh (2002) reported that RV myocyte loss from aging rats is increased due to mitochondrial release of cytochrome c leading to apoptosis. In contrast, Kajstura et al. (1996) demonstrated that DNA fragmentation differs between the LV and RV of aging rats and concluded that RV has less cell death. To complicate matters, myocyte loss may be sex-specific as well (Kessler et al., 2019).

Metabolic changes have also been reported in aging RV, with 24-month-old rat RVs demonstrating a differential activity of phosphofructokinase compared to LV (De Tata et al., 1988). Similarly, Anitha and Asha Devi (1996) reported that exercise altered glucose utilization in the RV in adult rats but not in aged rats. While these studies begin to illustrate and define how age alters RV function, mechanisms that drive the innate differences in the RV are lacking. Moreover, while it is clear that RV undergoes changes with aging, it is not known how these changes, in conjunction with disease, contribute to responses to therapies.

## Conclusion

It is becoming abundantly clear that the RV is functionally and molecularly unique from the LV. However, historically, little attention has been devoted to quantifying genetic, proteomic, and functional characteristics of the RV and accordingly, there is a paucity of information regarding the unique features of the RV. Complicating our understanding of chamber-specific differences is age-specific changes in hemodynamic stresses that the RV is subject to fetal development through adulthood and aging. Given the unique regulation and differences in response to therapies observed in the adult RV, it is imperative to also consider that age will also play a role in differential response to therapy.

## Author Contributions

Both authors listed have made a substantial, direct and intellectual contribution to the work, and approved it for publication.

### Conflict of Interest

The authors declare that the research was conducted in the absence of any commercial or financial relationships that could be construed as a potential conflict of interest.
